# Supplementary material for: Cuticular Chemistry of the Queensland Fruit Fly Bactrocera tryoni (Froggatt)
Source: Molecules. 2020 Sep 12;25(18):4185. doi: 10.3390/molecules25184185 (PMC7571174; doi:10.3390/molecules25184185)
Supplement: Supplementary file 1 [file molecules-25-04185-s001.pdf]

molecules

MDPI

Supplementary Materials

Cuticular Chemistry of the Queensland Fruit Fly *Bactrocera tryoni* (Froggatt)

Soo J. Park [1,2,*], Gunjan Pandey [1,3], Cynthia Castro-Vargas [1,3], John G. Oakeshott [1,3], Phillip W. Taylor [1,2] and Vivian Mendez [1,2]

[1] Applied BioSciences, Macquarie University, North Ryde NSW 2109, Australia; Gunjan.Pandey@csiro.au (G.P.); Cynthia.Castro-Vargas@csiro.au (C.C.-V.); John.Oakeshott@csiro.au (J.G.O.); phil.taylor@mq.edu.au (P.W.T.); vivian.mendez@mq.edu.au (V.M.)

[2] Australian Research Council Centre for Fruit Fly Biosecurity Innovation, Macquarie University, North Ryde, NSW 2109, Australia

[3] Commonwealth Scientific and Industrial Research Organisation Land and Water, Black Mountain, Acton, ACT 2601, Australia

* Correspondence: soojean.park@mq.edu.au; Tel.: +61-413-616-107

Received: 8 August 2020; Accepted: 10 September 2020; Published: 11 September 2020

Table of Contents

A. General Procedures..2
B. Synthesis of the amides...2
C. Mass spectra of methyl branched esters 4
References 6

A. General Procedures

^{1}H and ^{13}C Nuclear Magnetic Resonance (NMR) spectra were recorded using a Bruker Avance DPX 400 operating at 400 MHz for ^{1}H NMR and at 101 MHz for ^{13}C NMR. $CDCl_3$ was used as a solvent for all NMR samples. ^{1}H NMR chemical shifts are reported in parts per million (δ) referenced to the proton signal of the deuterated solvent ($CDCl_3$; 7.26 ppm), whereas ^{13}C NMR chemical shifts are reported with reference to the carbon signals of the deuterated solvent ($CDCl_3$: 77.16 ppm) unless otherwise stated. Shimadzu 2010 GCMS spectrometer was used to monitor reaction and to record low-resolution mass spectra with electron impact ionization(70 eV). Solvents were removed under reduced pressure using a Büchi Rotavapor R-200, Büchi V-500 vacuum pump, and Büchi B-490 heating bath set to a temperature of 40 °C. All reagents were purchased from Sigma-Aldrich, Merck, Ajax Finechem or Alfa-Aesar and used without further purification.

B. Synthesis of the amides

Amide synthesis was completed by the reaction of an amine with an acid anhydride in water, modified from a previously reported method. [1] In brief, To a solution of an amine (0.02 – 0.10 mol scale, 1 eq) in water (20 – 50 mL) was added a corresponding acid anhydride (1.1 eq). The reaction mixture was stirred at room temperature for 0.5 – 2 h. After the completion of a reaction monitored by GC, the aqueous reaction mixture was extracted with ethyl acetate (20 – 50 mL × 3). The organic layers were combined, washed with 5% $NaHCO_3$ solution (150 mL) and dried

over Na_2SO_4. The solvent was evaporated *in vacuo* to give the crude product, which was purified by vacuum distillation to give the pure product.

N-(3-Methylbutyl)acetamide. 0.5 h stirring, clear liquid, 7.2 g obtained (0.08 mol scale, 75% yield). ^{1}H NMR (400 MHz, $CDCl_3$) δ 0.86 (6 H, d, *J* = 6.6, CH(C**H**$_3$)$_2$), 1.34 (2 H, m, CH_2C**H**$_2$CH), 1.56 (1 H, sep, *J* = 6.7, CH_2C**H**(CH_3)$_2$), 1.92 (3 H, s, C**H**$_3$CO), 3.18 (2 H, m, NHC**H**$_2$), 6.21 (1 H, bs, N**H**); ^{13}C NMR (101 MHz, $CDCl_3$) δ 22.6, 23.3, 25.9, 38.1, 38.4, 170.5; GCMS (EI) *m/z* (%) 129 (M^+, 5), 114 (12), 86 (28), 73 ($CH_3CONHCH_3^+$, 100), 60 ($CH_3COHNH_2^{+\bullet}$, 38). Spectral data match with those in the literature.[2–3]

N-(2-Methylbutyl)propanamide. 1 h stirring, clear liquid, 2.23 g obtained (0.02 mol scale, 67% yield); ^{1}H NMR (400 MHz, $CDCl_3$) δ 0.83 (6 H, m, CH(CH_3)CH_2C**H**$_3$ and $COCH_2$C**H**$_3$), 1.09 (4 H, m, C**H**C**H**$_3$), 1.28 – 0.51 (2 H, m, CH(CH_3)C**H**$_2$$CH_3$), 2.17 (2 H, q, *J* = 7.6, CH_3C**H**$_2$CO), 2.95 – 3.15 (2 H, m, NHC**H**$_2$), 6.10 (1 H, bs, N**H**); ^{13}C NMR (101 MHz, $CDCl_3$) δ 10.0, 11.1, 17.0, 27.0, 29.6, 34.8, 45.0, 174.2; GCMS (EI) *m/z* (%) 143 (M^+, 10), 114 (26), 86 (75), 74 ($CH_3CH_2COHNH_2^{+\bullet}$, 61), 57 (100). Spectral data match with those in the literature.[2–3]

N-(3-Methylbutyl)propanamide. 2 h stirring, clear liquid, 11,3 g obtained (0.10 mol scale, 79% yield); ^{1}H NMR (400 MHz, $CDCl_3$) δ 0.80 (6 H, d, *J* = 6.6, CH(C**H**$_3$)$_2$), 1.04 (3 H, t, *J* = 7.6, C**H**$_3$$CH_2$O), 1.30 (2 H, q, *J* = 7.7, CH_2C**H**$_2$CH), 1.52 (1 H, apparent sep, *J* = 6.7, CH_2C**H**(CH_3)$_2$), 2.12 (2 H, q, *J* = 7.6, CH_3C**H**$_2$CO), 3.16 (2 H, q, *J* = 6.8, HNC**H**$_2$), 6.38 (1 H, bs, N**H**); ^{13}C NMR (101 MHz, $CDCl_3$) δ 10.1, 23.0, 25.4, 29.8, 36.8, 37.7, 174.7; GCMS (EI) *m/z* (%) 143 (M^+, 8), 128 (10), 114 (9), 100 (14), 87 ($CH_3CH_2CONHCH_3^+$, 86), 74 ($CH_3CH_2COHNH_2^{+\bullet}$, 36), 57 ($(CH_3)_2CHCH_2^+$, 100). Spectral data match with those in the literature.[2–3]

N-(3-methylbutyl)isobutyramide. 2 h, colourless needles, 6.83 g obtained (50 mmol scale, 87% yield). 1 h, clear liquid, 12.6 g obtained (0.10 mol scale, 87% yield. ^{1}H NMR (400 MHz, $CDCl_3$) δ 0.81 (6 H, d, *J* = 6.6, (C**H**$_3$)$_2$CHCO), 1.04 (6 H, d, *J* = 6.9, CH(C**H**$_3$)$_2$), 1.33 (2 H, apparent q, *J* = 7.5, CH_2C**H**$_2$CH), 1.53 (1 H, sep, *J* = 6.9, CH_2C**H**(CH_3)$_2$), 2.32 (1 H, sep, *J* = 6.6, (CH_3)$_2$C**H**CO), 3.17 (2 H, apparent q, *J* = 5.8, HNC**H**$_2$), 6.20 (1 H, bs, N**H**CH_2); ^{13}C NMR (100 MHz, $CDCl_3$) δ 19.6, 22.5, 25.9, 35.5, 37.7, 38.5, 177.2; GCMS (EI) *m/z* (%) 157 (M^+, 8), 142 (12), 114 (16), 101 ($(CH_3)_2CHCONH_2CH_2^+$, 50), 71 ((CH3)$_3$$CHCO^+$, 100). The spectral data match with those in the literature.[2–3]

C. Mass spectra of methyl branched fatty acid esters

2-Ethyl-8-methyl-1,7- dioxaspiro [5.5]undecane

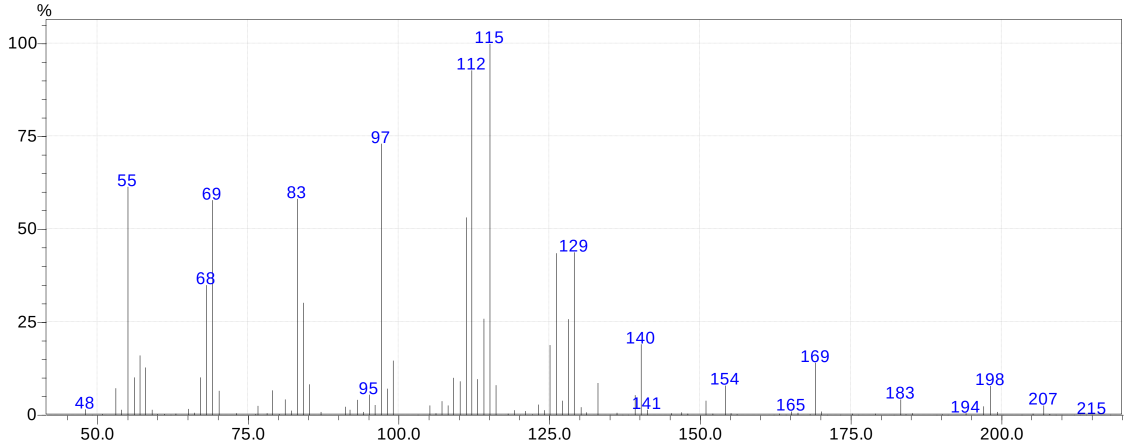

Ethyl 6-methyldodecanoate

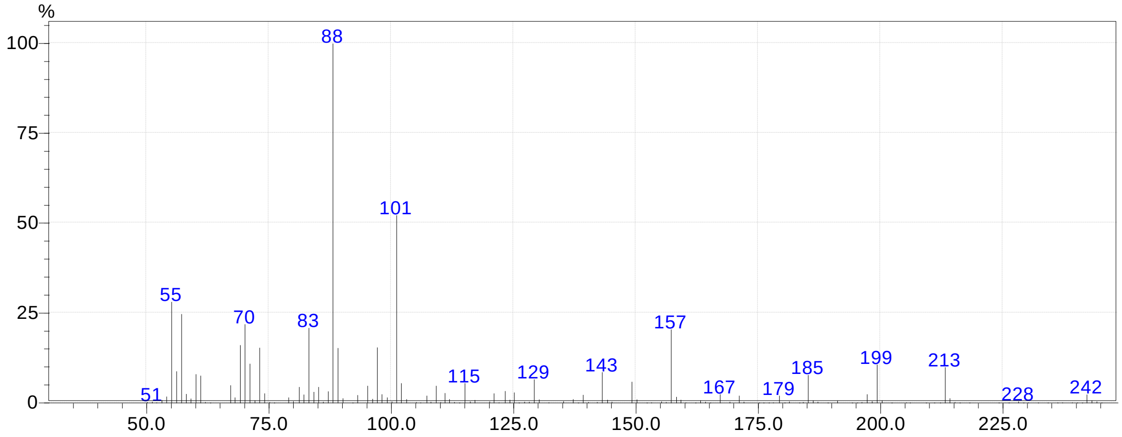

Ethyl 4-methyltetradecanoate

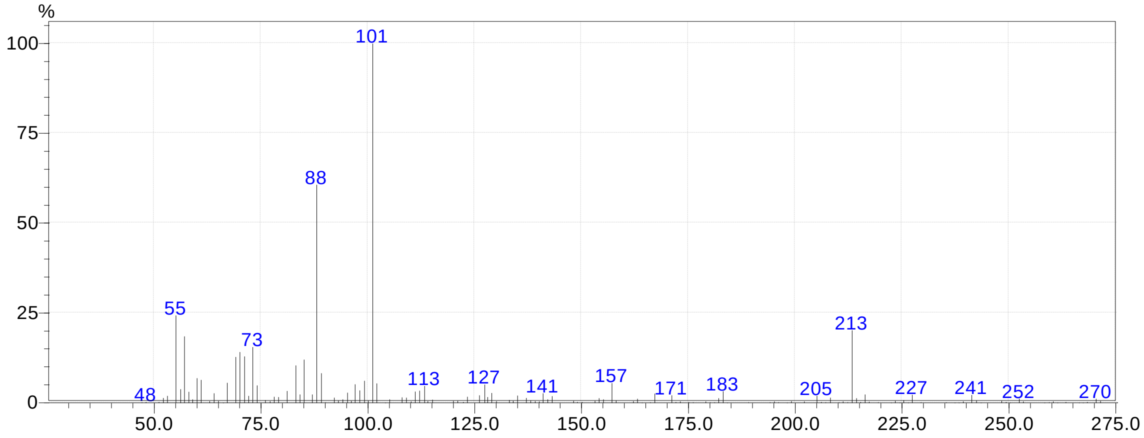

Ethyl 12-methyltetradecanoate

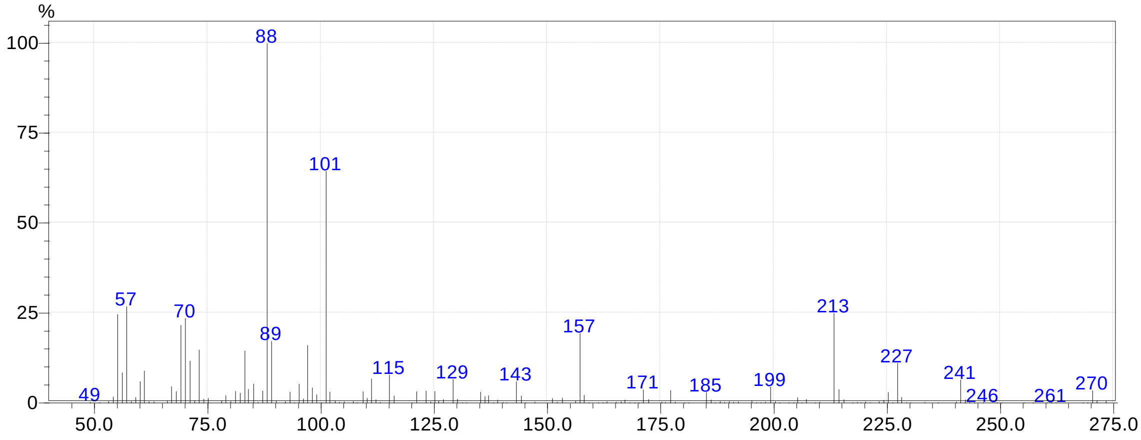

Ethyl 15-methylhexadecanoate

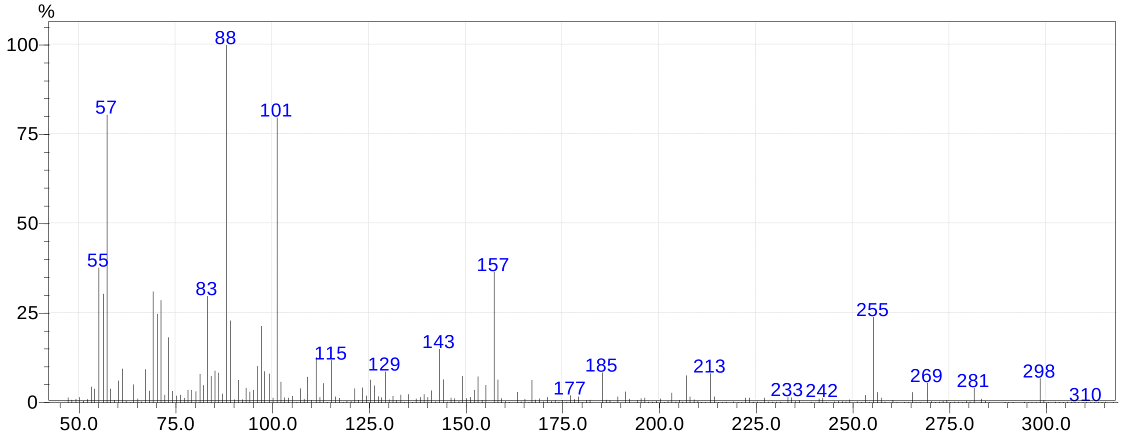

Ethyl 4-methylhexadecanoate

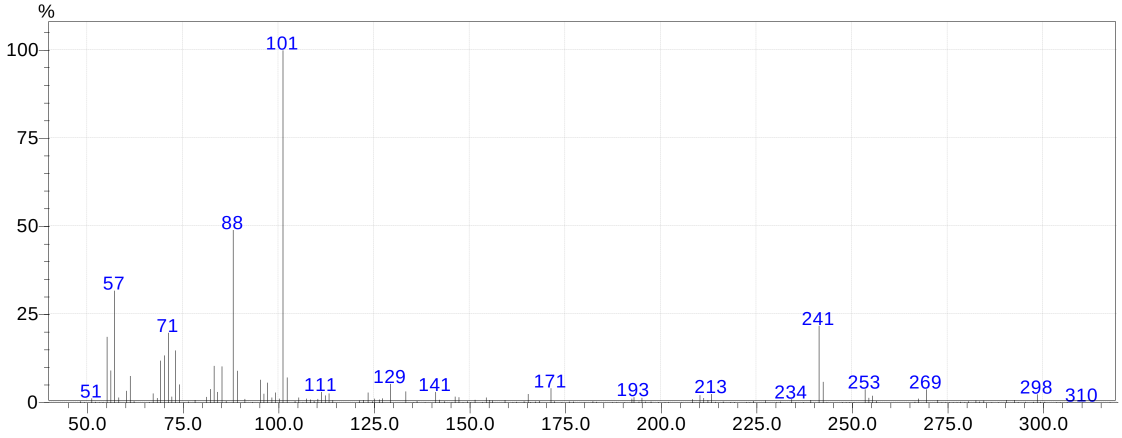

Ethyl 14-methylhexadecanoate

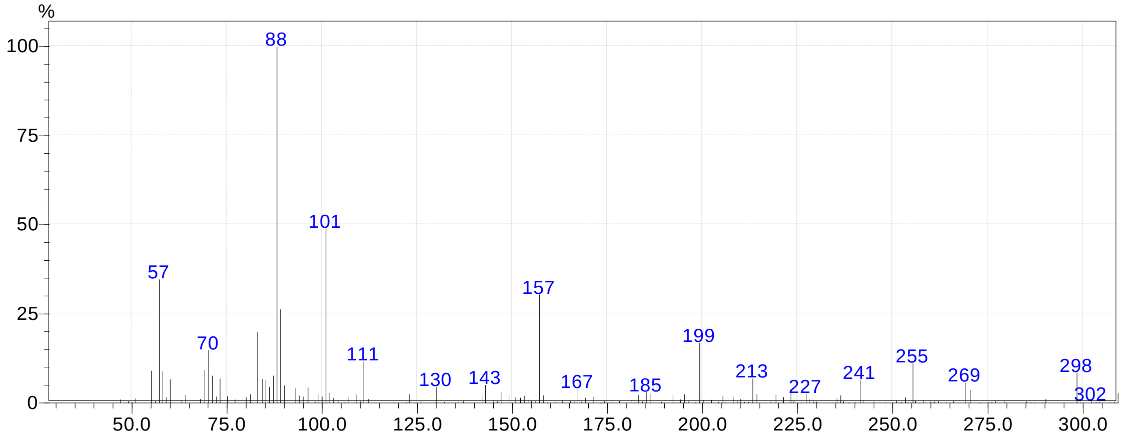

Propyl 9-hexadecenoic acid

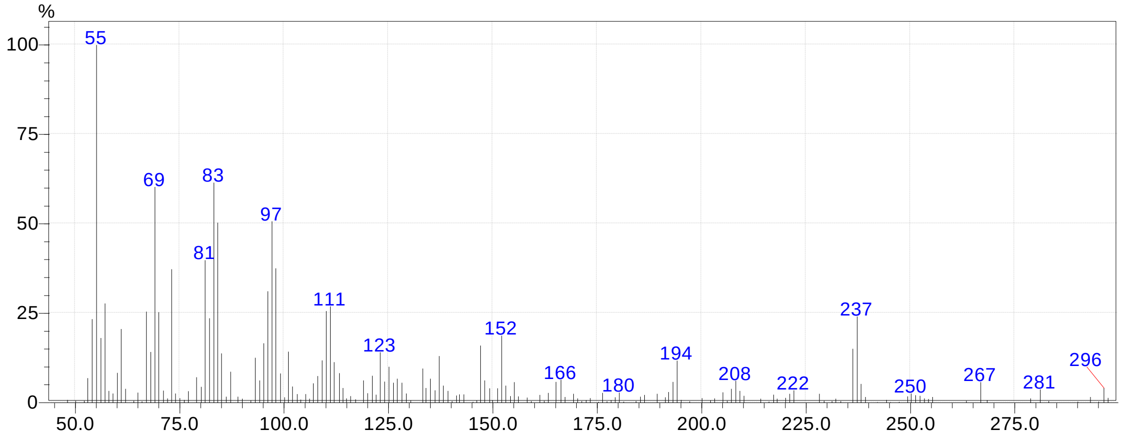

Ethyl 11-eicosenoate

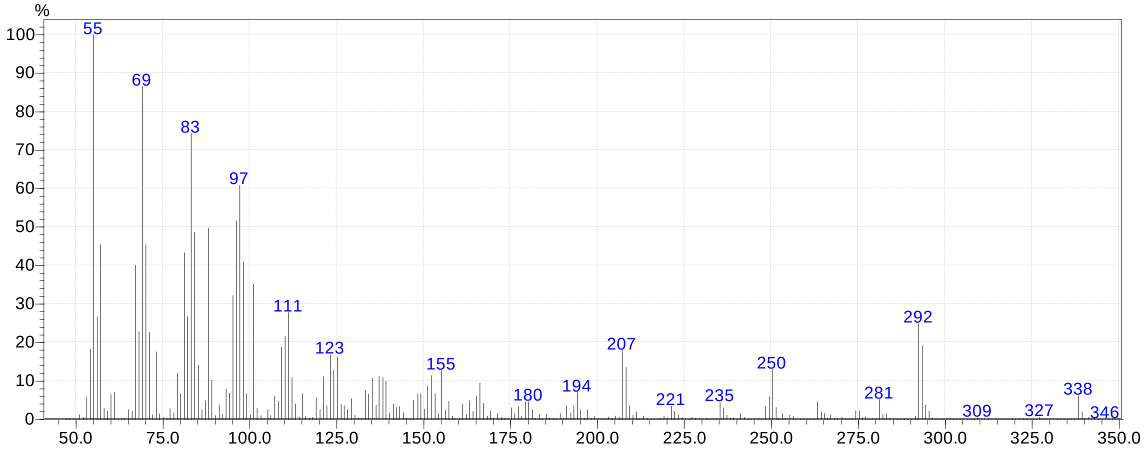

Figure S1. Mass spectra of 2-ethyl-8-methyl-1,7-dioxaspiro[5.5]undecane and the methyl branched fatty acid esters

References

1. Naik, S.; Bhattacharjya, G.; Talukdar, B.; Patel, Bhisma K., Chemoselective acylation of amines in aqueous media. *Eur. J. Org. Chem.* **2004,** *2004* (6), 1254-1260.
2. Pérez, J.; Park, S. J.; Taylor, P. W., Domestication modifies the volatile emissions produced by male Queensland fruit flies during sexual advertisement. *Sci. Rep.* **2018,** *8* (1), 16503.
3. Noushini, S.; Park, S. J.; Jamie, I. M.; Jamie, J. F.; Taylor, P. W., Sampling technique biases in the analysis of fruit fly pheromones: A case study of Queensland fruit fly. *Sci. Rep.* **2020,** *In press*.
